# Perceptions of the impact of individual allergic rhinitis symptoms: A survey of ARIA clinical experts

**DOI:** 10.1016/j.waojou.2024.100999

**Published:** 2024-12-03

**Authors:** Sara Gil-Mata, Tatiana Teixeira, Anna Bedbrook, Jean Bousquet, Bernardo Sousa-Pinto, Rafael José Vieira, ARIA group, Julijana Asllani, Habib Douagui, Estrella Asayag, Castro Maria Elizabeth, Carlos D. Crisci, René Maximiliano Gomez, Juan Carlos Ivancevich, Edgardo Jares, Jorge Fernando Máspero, Pablo Moreno, Hugo Eduardo Neffen, Mario Emilio Zernotti, Sinthia Bosnic-Anticevich, Kristin Chahhoud Carson, Biljana Cvetkovski, Janet M. Davies, Vicky Kritikos, Robyn O'Hehir, Brian Oliver, House Rachel Tan, Jessica Tattersall, Celia Zubrinich, Werner Aberer, Verena Niederberger-Leppin, Isabella Pali-Schöll, Virginie Doyen, Didier Ebo, Renaud Louis, Philippe Rombaux, Sophie Scheire, Kazi Bennoor, Bruno A. Barreto, Paulo Camargos, Herberto Jose Chong-Neto, Alvaro A. Cruz, Jane da Silva, Guidacci Marta, José Angelo Rizzo, Nelson Rosario Filho, Sarquis Serpa Faradiba, Dirceu Solé, Marilyn Urrutia-Pereira, George Christoff, Mandazhieva-Pepelanova Mariana, Odjakova Cvetanka, Todor A. Popov, Dilyana Vicheva, do Ceu Teixeira Maria, Jacques Bouchard, Jan Brozek, Derek K. Chu, Judah Denburg, Thomas Eiwegger, Paul K. Keith, Teresa To, Susan Waserman, Nancy Abusada, Emilio Alvarez Cuesta, Mario A. Calvo-Gil, Maria Antonieta Guzmán, Tamara Pérez Gomez, Wing Kin Wong Gary, Luo Zhang, Luis Caraballo, Alfonso Cepeda Sarabia, Dieudonné Nyembue, Manuel Soto-Martinez, Neven Miculinic, Davor Plavec, Constantinos Pitsios, Panayiotis Yiallouros, Petr Panzner, Milan Sova, Martina Vachova, Carsten Bindslev-Jensen, Ronald Dahl, Hans-Jorgen Malling, Lars Münter, Lars K. Poulsen, Suppli Ulrik Charlotte, Line Kring Tannert, Bassam Mahboub, Laila Salameh, Ivan Cherrez-Ojeda, de Guevara Karla Robles, Rasha Hassan El-Owaidy, Zeinab A. El-Sayed, Shereen Saad El-Sayed, Elham Hossny, Badr Eldin Mostafa, Kaja Julge, Nils Eric Billo, Patrik Eklund, Marina Erhola, Tari Haahtela, Jussi Karjalainen, Mika Makela, Sanna Toppila-Salmi, Erkka Valovirta, Tuula Vasankari, Isabella Annesi-Maesano, Isabelle Bossé, Melisande Bourgoin-Heck, Denis Charpin, André Coste, Frédéric de Blay, Philippe Devillier, Alain Didier, Anh Tuan Dinh-Xuan, Jean-François Fontaine, Jocelyne Just, Rachel Nadif, Nhân Pham-Thi, Bernard Pigearias, Nicolas Roche, Van Ganse Eric, Ekaterine Chkhartishvili, Amiran Gamkrelidze, Maia Gotua, Sven Becker, Karl-Christian Bergmann, Thomas Bieber, Randolf Brehler, Roland Buhl, Adam M. Chaker, Ulf Darsow, Eckard Hamelmann, Joachim Heinrich, Thomas Keil, Ludger Klimek, Pavel Kolkhir, Susanne Lau, Marcus Maurer, Ralph Mösges, Angelos Neou, Oliver Pfaar, Johannes Ring, Martin Wagenmann, Torsten Zuberbier, Aiste Ramanauskaite, Anja Lingnau, Demetrios Christou, Sophia Neisinger, Xenophon Aggelidis, Jannis Constantinidis, Maria Dimou, Christos Grigoreas, Trisevgeni Kapsali, Michael Katotomichelakis, Stelios Loukides, Michael Makris, Emmanouil Manousakis, Nikolaos Mikos, Aris Pagkalos, Nikolaos G. Papadopoulos, Vasileios Papanikolaou, Giannis Paraskevopoulos, Konstantina Piskou, Emmanuel Prokopakis, Fotios Psarros, Konstantinos Samitas, Sofia Stamataki, Evangelia Stefanaki Lina, Ekaterini Syrigou, Ioanna Tsiligianni, Mina Vallianatou, Dimitrios Vourdas, Paraskevi Xepapadaki, Aspasia Karavelia, Fanny Wai San Ko, Gary Wong, Andor Hirschberg, Helga Kraxner, Kristof Nekam, Stephen Lane, Menachem Rottem, Cristina Artesani Maria, Simona Barbaglia, Bianca Beghé, Beatrice Bilo Maria, Attilio Boner, Matteo Bonini, Sergio Bonini, Fulvio Braido, Luisa Brussino, G Walter Canonica, Lorenzo Cecchi, Giorgio Ciprandi, Enrico Compalati, Gennaro D'Amato, Giulia De Feo, Stefano Del Giacco, Alessandro Fiocchi, Enrico Heffler, Carlo Lombardi, Riccardo Monti, Antonella Muraro, Eustachio Nettis, Stefania Nicola, Giovanni Passalacqua, Vincenzo Patella, Francesca Puggioni, Giovanni Rolla, Antonino Romano, Nicola Scichilone, Massimo Triggiani, Teresa Ventura Maria, Giovanni Viegi, Cristina Boccabella, Mattia Giovannini, Anand Mahesh Padukudru, Tomohisa Iinuma, Ken Ohta, Yoshitaka Okamoto, Kimihiro Okubo, Ruby Pawankar, Daiju Sakurai, Maho Suzukawa, Masao Yamaguchi, Zhanat Ispayeva, Yoon-Seok Chang, Hae-Sim Park, Mona Al-Ahmad, Ieva Cirule, Ineta Grisle, Carla Irani, Philip Rouadi, Fares Zaitoun, Ruta Dubakiene, Regina Emuzyte, Violeta Kvedariene, Brigita Gradauskiene, Arunas Valiulis, Markus Ollert, Farah Hannachi, Dejan Dokic, Amir Hamzah Abdul Latiff, Baharudin Abdullah, S.P. Palaniappan, Kent Woo, Stephen Montefort, Martín Bedolla Barajas, María del Carmen Costa Domínguez, Jesús Guillermo Espinoza Contreras, Jose Miguel Fuentes Pèrez, José Luis Gálvez Romero, María de la Luz Hortensia García Cruz, Sandra González Diaz, Yunuen Rocío Huerta Villalobos, Désiree E. Larenas-Linnemann, Jorge Agustin Luna-Pech, Juan José Matta Campos, Daniela Rivero Yeverino, Mónica Rodríguez González, Eréndira Rodríguez Zagal, Battur Lkhagvaa, Sandra Mavale-Manuel, Abrantes Nunes Elizabete, Niels Chavannes, Wytske J. Fokkens, Gerard Koppelman, Anke-Hilse Maitland-van der Zee, Sietze Reitsma, Ramanathan Chandrasekharan, Osman Yusuf, Maria Susana Repka-Ramirez, Juan Carlos Sisul, José E. Gereda, Marysia T. Recto, Slawomir Bialek, Artur Bialoszewski, Marta Chelminska, Radoslaw Gawlik, Ewa Jassem, Marek Jutel, Piotr Kuna, Maciej Kupczyk, Marcin Moniuszko, Marek Niedoszytko, Filip Raciborski, Jan Romantowski, Boleslaw Samolinski, Krzysztof Specjalski, Pedro Carreiro Martins, Jaime Correia de Sousa, Elisio Costa, Joao A. Fonseca, Olga Lourenço, Mario Morais-Almeida, Margarida Pereira Ana, Frederico Regateiro, Carlos Robalo Cordeiro, Jose Rosado Pinto, Bernardo Sousa-Pinto, Luis Taborda Barata, Ana Maria Todo-Bom, José Vieira Rafael, Daniela Carvalho, Maryam Ali Al-Nesf, Ioana Agache, Camelia Berghea Elena, Roxana Bumbacea, Diana Deleanu, Florin Mihaltan, Carmen Panaitescu Bunu, Musa Khaitov, Leyla Namazova-Baranova, Elena Vishneva, Ali Alshaikh Nada, Sanja Dimic-Janjic, Branislava Milenkovic, De Yun Wang, Martin Hrubiško, Natalija Edelbaher, Maja Jošt, Peter Kopac, Mitja Košnik, Nika Lalek, Antonija Poplas Susic, Irma Rozman Sinur, Tanja Soklic Košak, Nadja Triller, Katja Triller, Jure Urbancic, Mihaela Zidarn, Michael Levin, Paul Potter, Heather Zar, Isam Alobid, Aram Anto, Joan Bartra, Irina Bobolea, Paloma Campo, Vicky Cardona, Maria Carriazo Ana, José Antonio Castillo Vizuete, Tomas Chivato, Ignacio Jesus Davila Gonzalez, Joaquim Mullol, Antonio Nieto Garcia, César Picado, Santiago Quirce, Joaquin Sastre, Leticia de las Vecillas, Mikael Benson, Inger Kull, Marianne van Hage, Magnus Wickman, Nikolai Khaltaev, Yousser Mohammad, Pongsakorn Tantilipikorn, Pakit Vichyanond, Cemal Cingi, Bilun Gemicioglu, Ozlem Goksel, Omer Kalayci, Ali Fuat Kalyoncu, Cem Meço, Koyuncu Ilgim Vardaloglu, Arzu Yorgancioglu, Deniz Eyice Karabacak, Bruce Kirenga, Ian Michael Adcock, Hasan S. Arshad, Mike Bewick, Christine Bond, Christopher Brightling, Andrew Bush, Moïses Calderon, Kian Fan Chung, Adnan Custovic, Ratko Djukanovic, Stephen Durham, John Farrell, David Halpin, Michael Hyland, Sebastian Johnston, Brian Lipworth, Alla Nakonechna, David Price, Graham Roberts, Dermot Ryan, Jürgen Schwarze, Aziz Sheikh, Mike Shields, Samantha Walker, Sian Williams, Igor Kaidashev, Andrii Igorevich Kurchenko, Vladyslav Tsaryk, David Bernstein, Jonathan A. Bernstein, Thomas B. Casale, Sharon Chinthrajah, Mark Dykewicz, Gailen D. Marshall, Eli O. Meltzer, Robert M. Naclerio, Alkis Togias, Elina Toskala, Dana Wallace, Dennis M. Williams, Barbara Yawn, Fernan Caballero-Fonseca, Lan Le Thi Tuyet, Tran Thien Quan Vu

**Affiliations:** a Department of Community Medicine, Information and Health Decision Sciences (MEDCIDS), Faculty of Medicine of the University of Porto, Porto, Portugal; b CINTESIS@RISE — Center for Health Technology and Services Research, Health Research Network, Porto, Portugal; c ARIA, Montpellier, France; d Institute of Allergology, Charite Universitätsmedizin Berlin, Corporate Member of Freie University at Berlin and Humboldt-Universtät zu Berlin, Berlin, Germany; e Fraunhofer Institute for Translational Medicine and Pharmacology ITMP, Immunology and Allergology, Berlin, Germany

**Keywords:** Allergic rhinitis, Survey, Professional-patient relation

## Abstract

**Background:**

Allergic rhinitis (AR) is a highly prevalent disease. We aimed to assess the symptoms that physicians who see patients with AR perceive as the most bothersome in their patients.

**Methods:**

We performed a cross-sectional study based on an online questionnaire sent to all members of the Allergic Rhinitis and its Impact on Asthma (ARIA) initiative. The survey included questions on the physicians' perceptions of patients’ AR symptoms as well as of their own AR symptoms.

**Results:**

Among 401 respondents, 155 (38.7%) reported having AR. ARIA members reported nasal symptoms to be the most frequent (89.7%) and bothersome (80.0%) symptoms experienced by themselves. Likewise, nasal symptoms were reported by ARIA members as the most frequent (94.8% in members with AR vs 96.0% in members without AR) and bothersome (57.0% in members with AR vs 67.9% in members without AR) in their patients. We found a significant association (p = 0.001) between physicians’ own symptoms and those perceived as the most bothersome in their patients.

**Conclusion:**

Physicians perceive nasal symptoms to be the most frequent and the most bothersome symptoms in AR patients. The physicians' personal experiences with AR may influence their perception of patients’ symptoms.

## Introduction

Patients with allergic rhinitis (AR) present with nasal symptoms^1^ and, frequently, with ocular and lower respiratory symptoms.^2^^,^^3^ These symptoms may impact AR patients differently, according to the severity of the disease and to patients’ perceptions of values and preferences (V&Ps).

Healthcare interventions typically result in benefits and harms. Patients' values and preferences concern the relative importance patients place on specific benefits and harms. At a group level, considering V&Ps are essential for patient-centred guidelines, particularly as guidelines typically deal with multiple outcomes which may be differently affected by interventions. In this context, a systematic review on patients’ V&Ps in allergic rhinitis found that patients considered (i) nasal symptoms as more important than ocular ones and (ii) nasal obstruction as the most impactful nasal symptom (even though the certainty of the evidence tended to be low).^4^ This systematic review at the group level can be complemented by other studies with different approaches.

At the individual patient level, in the context of daily clinical practice, it is also relevant to consider each patient's perspective on which AR symptoms have the highest impact. However, individual patient perceptions may not always align with those of the physician. Indeed, such discrepancies between healthcare professionals and patients in their perceptions of disease have been reported in chronic inflammatory disorders, namely systemic sclerosis and psoriasis.^5^^,^^6^ This may be particularly important in a highly prevalent disease such as AR, as physicians often present AR themselves.^7^ Physicians' personal experiences with their own disease, including its symptoms and their impact, may shape their perception of their patients' disease manifestations.

The main aim of this study was to assess the symptoms that ARIA members who see patients with AR perceive as the most bothersome in their patients. As a secondary endpoint, we assessed whether there was an association between the most frequent and bothersome symptoms reported by physicians who suffer from AR and their perception of patients’ symptoms. Although this study does not formally elicit values and preferences, it may partly complement the results of the systematic review that addressed them.

## Methods

We conducted a cross-sectional study based on an online structured questionnaire sent by email to all registered Allergic Rhinitis and its Impact on Asthma (ARIA) initiative members. ARIA is a network of experts on AR and asthma, including healthcare professionals and researchers.^8^

The survey was anonymous and included 3 different sections (Supplemental Box 1). The first section inquired about demographic and professional information. Two additional questions in this section asked whether the respondents managed patients with AR in their clinical practice and whether the respondents had AR themselves. The second section targeted physicians who managed patients with AR. There were 3 questions: (i) which symptoms were reported as the most frequent by their patients with AR (nasal, ocular, asthma, or other); (ii) their perception of which symptoms were described as the most bothersome by their patients with AR (nasal, ocular, asthma, or other), and (iii) specifically, what was the most bothersome nasal symptom (nasal congestion, itching, rhinorrhea, or sneezing). For respondents who reported having AR, a third section encompassed questions on physicians’ own symptoms.

A follow-up email was sent as a reminder to improve response rates. The questionnaire was sent to 699 respondents. Responses were collected between 8 November and 31 December, 2023.

We assessed whether physicians with AR differed from those without AR on the symptoms they perceive as the most frequent and bothersome in their patients. Additionally, we assessed, among respondents who have AR, whether there is an association between physicians’ own symptoms and their perception of symptoms reported by their AR patients. Finally, we studied the association between age and sex of ARIA members with AR and their most frequent and bothersome symptoms.

Categorical variables were described using absolute and relative frequencies. Chi-square or Fisher's exact test were used to test for the association between variables.

## Results

**Fig. 1:**
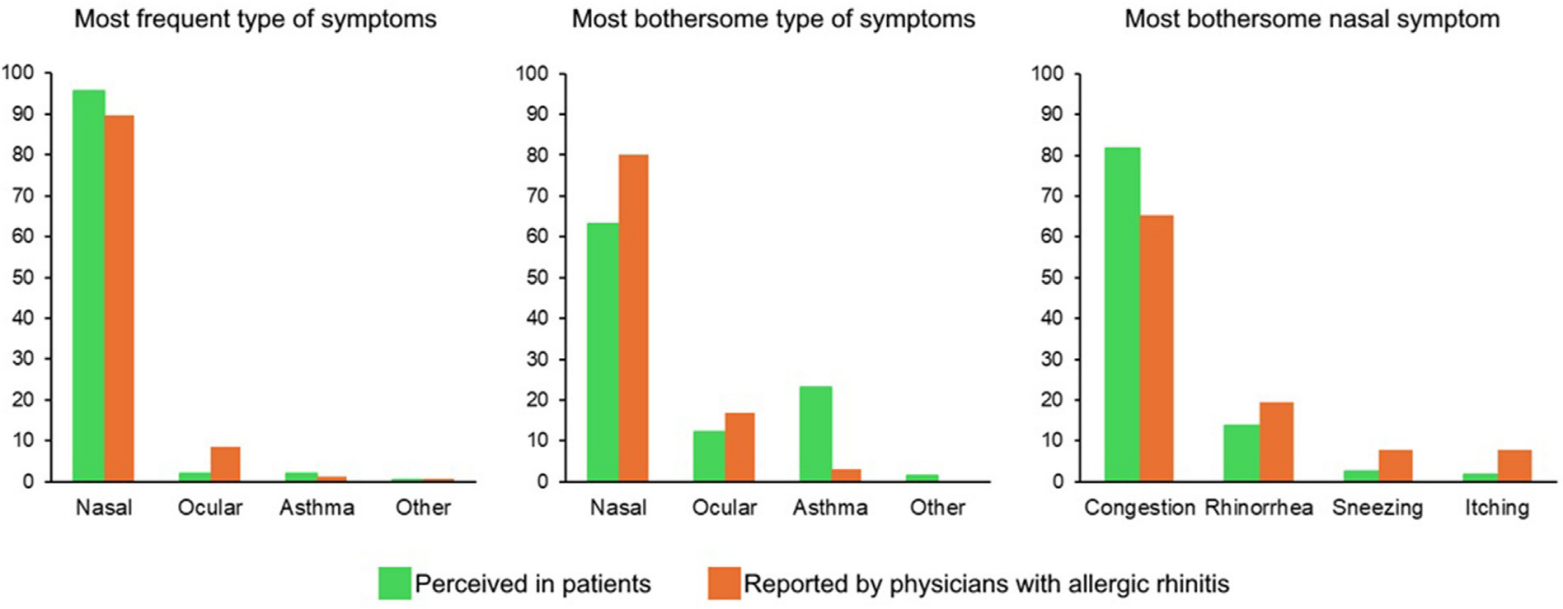
Most frequent (A) and most bothersome (B and C) allergic rhinitis symptoms perceived in patients by ARIA members and reported by ARIA members.

A total of 401 responses were received (57.4% of ARIA members); 254 respondents (63.3%) were from Europe (Table 1); and 47.9% respondents were aged between 51 and 65 years (Table 1). Most respondents were physicians (90.8%) and, among these, 248 (68.1%) reported having more than 25 years of clinical practice. AR was reported by 155 (38.7%) ARIA members and, among those, 135 (87.1%) respondents reported seeing patients with AR. According to the ARIA classification, most members reported having mild intermittent AR (50.3%), followed by mild persistent (25.8%), moderate-severe persistent (12.9%), and moderate-severe intermittent rhinitis (11.0%). ARIA members with AR reported nasal symptoms to be their most frequent (89.7%) and bothersome (80.0%) symptoms (Fig. 1A and B). Among nasal symptoms, nasal congestion was considered to be the most bothersome (65.2%), followed by rhinorrhea (19.4%) (Fig. 1C).

**Table 1:** Demographics, clinical characteristics and professional profile of survey respondents (n = 401)

18–35 years	14 (3.5)
**(i) Age – N (%)**	
18–35 years	14 (3.5)
36–50 years	79 (19.7)
51–65 years	192 (47.9)
66 or more years	109 (27.2)
Not answered	7 (1.7)
**(ii) Sex – N (%)**	
Female	149 (37.2)
Male	251 (62.6)
Prefer not to say	1 (0.2)
**(iii) Location – N (%)**	
Africa	14 (3.5)
Asia	53 (13.2)
Europe	254 (63.3)
North America	36 (9.0)
Oceania	9 (2.2)
South America	35 (8.7)
**(iv) Suffers from AR – N (%)**	
Yes	155 (38.7)
No	235 (58.6)
Prefer not to say	11 (2.7)
**(v) ARIA classification – N (%)**	
Mild intermittent	78 (50.3)
Mild persistent	40 (25.8)
Moderate-severe intermittent	17 (11.0)
Moderate-severe persistent	20 (12.9)
**(vi) Specialty – N (%)**	
Allergy/Allergology	155 (38.7)
General practice/Primary care	11 (2.7)
Internal medicine	46 (11.5)
Otorhinolaryngology (ENT physician)	44 (11.0)
Paediatrics	68 (17.0)
Pulmonology	95 (23.7)
Other or not a physician	47 (11.7)
**(vii) Years of clinical practice – N (%)**	
Less than 10	17 (4.7)
10–25	88 (24.2)
More than 25	248 (68.1)
Not in clinical practice	11 (3.0)
**(viii) Sees patients with AR – N (%)**	
Yes	340 (84.8)

Overall, the symptoms reported by allergic and non-allergic ARIA members as the most frequent in their patients were nasal symptoms (94.8% in members with AR vs 96.0% in members without AR) (Supplemental Table 1A). Nasal symptoms were indicated as the most bothersome to patients (57.0% in members with AR vs 67.9% in members without AR) (Supplemental Table 1B). Asthma was identified as the most bothersome symptom in patients by 25.2% of ARIA members with AR and 21.0% of ARIA members without AR. Among nasal symptoms, nasal congestion was perceived as the most bothersome in patients (according to 83.7% of ARIA members with AR and 82.1% of ARIA members without AR) (Supplemental Table 1C).

The most bothersome set of symptoms experienced by physicians suffering from AR was significantly associated with those perceived as the most bothersome to their patients (*p* = 0.001) (Supplemental Table 2). The same was not observed for the most frequent set of symptoms experienced by physicians suffering from AR (Supplemental Table 3). No significant association was found between the presence vs absence of AR in physicians and their perception of the most frequent and bothersome patients’ symptoms.

Similarly, there were no significant associations between the age or sex of physicians and their most bothersome (Supplemental Table 2) or frequent (Supplemental Table 3) set of symptoms.

## Discussion

In this survey, we found that physicians (whether or not they suffer from AR) perceive nasal symptoms to be the most frequent and the most bothersome symptoms among their AR patients.

In a recent systematic review on AR patients' values and preferences, nasal symptoms were identified as the most frequent manifestation of AR, with nasal congestion being reported as the most frequent and the most bothersome symptom.^4^ Nevertheless, in 3 out of 13 included studies, and in 5 out of 8 studies, an ocular and a non-nasal respiratory symptom, respectively, were identified as the most or second most important symptom.^4^ This study suggests that physicians’ perceptions on the most bothersome symptom in patients with AR may be in line with the results of the systematic review.

Interestingly, there were no differences between age groups and sex regarding symptoms considered the most frequent and bothersome by ARIA members suffering from AR. Although there is some evidence that a different symptom profile may be associated with those variables,^9^, ^10^, ^11^, ^12^ such was not observed in our study. In fact, ARIA members may have more sociodemographic similarities among themselves (not being fully representative of patients with AR) and more self-awareness of their disease than patients included in other studies, which may partly explain our findings.

There are some limitations to this study. Firstly, the sample was exclusively composed of ARIA members, excluding healthcare professionals who are not affiliated with the ARIA initiative. This may introduce a bias in our study due to the fact that allergists, who usually see more severe cases of AR, are overrepresented in the ARIA group.

In addition, demographic characteristics of ARIA members may raise concerns on the generalizability of our results. There is an overrepresentation of physicians older than 50 and, although we found no evidence of an association between symptoms and age, a different symptom profile has been previously reported in older individuals with AR.^12^ Our survey did not specifically inquire about the physicians' perception of paediatric patient-reported symptoms. Since in the context of paediatric care it is common that a caregiver intervenes in children's symptom reporting, it is possible that the influence of the physician's perspective may differ in this subgroup. Finally, other limitations of our study include possible information biases arising from errors when filling in the form.

Our study has also some strengths. To the best of our knowledge, this is the first study to address whether the physicians' own experiences as AR patients influence their perceptions on their patients' symptoms. Our results suggest that patient-reported outcome measures may be of particular importance in the clinical assessment of highly prevalent diseases as a means to adequately incorporate patients’ V&Ps in the management of their disease and to enhance communication between both parts.

In conclusion, our study reveals that nasal symptoms, particularly nasal congestion, are the most frequent and the most bothersome symptoms, not only as perceived by physicians when evaluating their patients, but also as experienced by themselves as AR patients. Furthermore, our results suggest a possible association between the physicians' personal experiences with AR and their perception of patients’ symptoms. Despite some limitations, the present study provides a valuable insight into patient-physician interactions in the context of a highly prevalent disease.

## Author contribution statement

Sara Gil-Mata: Data analysis and interpretation, Drafting the article. Tatiana Teixeira: Data analysis and interpretation, Drafting the article. Anna Bedbrook: Data collection, Critical revision of the article. Jean Bousquet: Conception, Data collection, Critical revision of the article. Bernardo Sousa-Pinto: Conception, Data collection, Critical revision of the article. Rafael José Vieira: Conception, Data collection, Critical revision of the article.

## Availability of data and materials

The dataset that supports the findings of this study is available from the corresponding author upon reasonable request.

## Authors’ consent for publication

All authors have provided consent for the publication of the present manuscript.

## Ethics approval

Not applicable.

## Funding sources

This work has received funding from: ARIA (Allergic Rhinitis and its Impact of Asthma), Montpellier, France; CINTESIS, University of Porto, Porto, Portugal.

## Declaration of competing interest

The authors report no competing interests.

## Appendix ARIA group:

Asllani Julijana from Albania, Douagui Habib from Algeria, Asayag Estrella, Castro Maria Elizabeth, Crisci Carlos D, Gomez René Maximiliano, Ivancevich Juan Carlos, Jares Edgardo, Máspero Jorge Fernando, Moreno Pablo, Neffen Hugo Eduardo, Zernotti Mario Emilio from Argentina, Bosnic-Anticevich Sinthia, Chahhoud Carson Kristin, Cvetkovski Biljana, Davies Janet M, Kritikos Vicky, O’Hehir Robyn, Oliver Brian, Tan House Rachel, Tattersall Jessica, Zubrinich Celia from Australia, Aberer Werner, Niederberger-Leppin Verena, Pali-Schöll Isabella from Austria, Doyen Virginie, Ebo Didier, Louis Renaud, Rombaux Philippe, Scheire Sophie from Belgium, Kazi Bennoor from Bengladesh, Barreto Bruno A, Camargos Paulo, Chong-Neto Herberto Jose, Cruz Alvaro A, da Silva Jane, Guidacci Marta, Rizzo José Angelo, Rosario Filho Nelson, Sarquis Serpa Faradiba, Solé Dirceu, Urrutia-Pereira Marilyn from Brazil, Christoff George, Mandazhieva-Pepelanova Mariana, Odjakova Cvetanka, Popov Todor A, Vicheva Dilyana from Bulgaria, do Ceu Teixeira Maria from Cabo Verde, Bouchard Jacques, Brozek Jan, Chu Derek K, Denburg Judah, Eiwegger Thomas, Keith Paul K, To Teresa, Waserman Susan from Canada, Abusada Nancy, Alvarez Cuesta Emilio, Calvo-Gil Mario A, Guzmán Maria Antonieta, Pérez Gomez Tamara from Chile, Wing Kin Wong Gary, Zhang Luo from China, Caraballo Luis, Cepeda Sarabia Alfonso from Colombia, Nyembue Dieudonné from Congo, Soto-Martinez Manuel from Costa Rica, Miculinic Neven, Plavec Davor from Croatia, Pitsios Constantinos, Yiallouros Panayiotis from Cyprus, Panzner Petr, Sova Milan, Vachova Martina from Czech Republic, Bindslev-Jensen Carsten, Dahl Ronald, Malling Hans-Jorgen, Münter Lars, Poulsen Lars K, Suppli Ulrik Charlotte, Tannert Line Kring from Denmark, Mahboub Bassam, Salameh Laila from United Arab Emirates, Cherrez-Ojeda Ivan, Robles de Guevara Karla from Ecuador, El-Owaidy Rasha Hassan, El-Sayed Zeinab A, El-Sayed Shereen Saad, Hossny Elham, Mostafa Badr Eldin from Egypt, Julge Kaja from Estonia, Billo Nils Eric, Eklund Patrik, Erhola Marina, Haahtela Tari, Karjalainen Jussi, Makela Mika, Toppila-Salmi Sanna, Valovirta Erkka, Vasankari Tuula from Finland, Annesi-Maesano Isabella, Bossé Isabelle, Bourgoin-Heck Melisande, Charpin Denis, Coste André, de Blay Frédéric, Devillier Philippe, Didier Alain, Dinh-Xuan Anh Tuan, Fontaine Jean-François, Just Jocelyne, Nadif Rachel, Pham-Thi Nhân, Pigearias Bernard,Roche Nicolas, Van Ganse Eric from France, Chkhartishvili Ekaterine, Gamkrelidze Amiran, Gotua Maia from Georgia, Becker Sven, Bergmann Karl-Christian, Bieber Thomas, Brehler Randolf, Buhl Roland, Chaker Adam M, Darsow Ulf, Hamelmann Eckard, Heinrich Joachim, Keil Thomas, Klimek Ludger, Kolkhir Pavel, Lau Susanne, Maurer Marcus, Mösges Ralph, Neou Angelos, Pfaar Oliver, Ring Johannes, Wagenmann Martin, Zuberbier Torsten, Ramanauskaite Aiste, Lingnau Anja, Christou Demetrios, Neisinger Sophia from Germany, Aggelidis Xenophon, Constantinidis Jannis, Dimou Maria, Grigoreas Christos, Kapsali Trisevgeni, Katotomichelakis Michael, Loukides Stelios, Makris Michael, Manousakis Emmanouil, Mikos Nikolaos, Pagkalos Aris, Papadopoulos Nikolaos G, Papanikolaou Vasileios, Paraskevopoulos Giannis, Piskou Konstantina, Prokopakis Emmanuel, Psarros Fotios, Samitas Konstantinos, Stamataki Sofia, Stefanaki Lina Evangelia, Syrigou Ekaterini, Tsiligianni Ioanna, Vallianatou Mina, Vourdas Dimitrios, Xepapadaki Paraskevi, Karavelia Aspasia from Greece, Wai San Ko Fanny, Wong Gary from Hong Kong, Hirschberg Andor, Kraxner Helga, Nekam Kristof from Hungary, Lane Stephen from Ireland, Rottem Menachem from Israel, Artesani Maria Cristina, Barbaglia Simona, Beghé Bianca, Bilo Maria Beatrice, Boner Attilio, Bonini Matteo, Bonini Sergio, Braido Fulvio, Brussino Luisa, Canonica G Walter, Cecchi Lorenzo, Ciprandi Giorgio, Compalati Enrico, D'Amato Gennaro, De Feo Giulia, Del Giacco Stefano, Fiocchi Alessandro, Heffler Enrico, Lombardi Carlo, Monti Riccardo, Muraro Antonella, Nettis Eustachio, Nicola Stefania, Passalacqua Giovanni, Patella Vincenzo, Puggioni Francesca, Rolla Giovanni, Romano Antonino, Scichilone Nicola, Triggiani Massimo, Ventura Maria Teresa, Viegi Giovanni, Boccabella Cristina, Giovannini Mattia from Italy, Mahesh Padukudru Anand from India, Iinuma Tomohisa, Ohta Ken, Okamoto Yoshitaka, Okubo Kimihiro, Pawankar Ruby, Sakurai Daiju, Suzukawa Maho, Yamaguchi Masao from Japan, Ispayeva Zhanat from Kazakhstan, Chang Yoon-Seok, Park Hae-Sim from Korea, Al-Ahmad Mona from Kuwait, Cirule Ieva, Grisle Ineta from Latvia, Irani Carla, Rouadi Philip, Zaitoun Fares from Lebanon, Dubakiene Ruta, Emuzyte Regina, Kvedariene Violeta, Gradauskiene Brigita, Valiulis Arunas fom Lithuania, Ollert Markus, Hannachi Farah from Luxemburg, Dokic Dejan from Macedonia, Abdul Latiff Amir Hamzah, Abdullah Baharudin, Palaniappan SP, Woo Kent from Malaysia, Montefort Stephen from Malta, Bedolla Barajas Martín, Costa Domínguez María del Carmen, Espinoza Contreras Jesús Guillermo, Fuentes Pèrez Jose Miguel, Gálvez Romero José Luis, García Cruz María de la Luz Hortensia, González Diaz Sandra, Huerta Villalobos Yunuen Rocío, Larenas-Linnemann Désiree E, Luna-Pech Jorge Agustin, Matta Campos Juan José, Rivero Yeverino Daniela, Rodríguez González Mónica, Rodríguez Zagal Eréndira from Mexico, Lkhagvaa Battur from Mongolia, Mavale-Manuel Sandra, Nunes Elizabete Abrantes from Mozambique, Chavannes Niels, Fokkens Wytske J, Koppelman Gerard, Maitland-van der Zee Anke-Hilse, Reitsma Sietze from the Netherlands, Chandrasekharan Ramanathan from Oman, Yusuf Osman from Pakistan, Repka-Ramirez Maria Susana, Sisul Juan Carlos from Paraguay, Gereda José E from Peru, Recto Marysia T from the Philippines, Bialek Slawomir, Bialoszewski Artur, Chelminska Marta, Gawlik Radoslaw, Jassem Ewa, Jutel Marek, Kuna Piotr, Kupczyk Maciej, Moniuszko Marcin, Niedoszytko Marek, Raciborski Filip, Romantowski Jan, Samolinski Boleslaw, Specjalski Krzysztof from Poland, Carreiro Martins Pedro, Correia de Sousa Jaime, Costa Elisio, Fonseca Joao A, Lourenço Olga, Morais-Almeida Mario, Pereira Ana Margarida, Regateiro Frederico, Robalo Cordeiro Carlos, Rosado Pinto Jose, Sousa-Pinto Bernardo, Taborda Barata Luis, Todo-Bom Ana Maria, Vieira Rafael José, Carvalho Daniela from Portugal, Al-Nesf Maryam Ali from Qatar, Agache Ioana, Berghea Elena Camelia, Bumbacea Roxana, Deleanu Diana, Mihaltan Florin, Panaitescu Bunu Carmen from Romania, Khaitov Musa, Namazova-Baranova Leyla, Vishneva Elena from Russia, Alshaikh Nada Ali from Saudi Arabia, Dimic-Janjic Sanja, Milenkovic Branislava from Serbia, Wang De Yun from Singapore, Hrubiško Martin from Slovakia, Edelbaher Natalija, Jošt Maja, Kopac Peter, Košnik Mitja, Lalek Nika, Poplas Susic Antonija, Rozman Sinur Irma, Soklic Košak Tanja, Triller Nadja, Triller Katja, Urbancic Jure, Zidarn Mihaela from Slovania, Levin Michael, Potter Paul, Zar Heather from South Africa, Alobid Isam, Anto Aram, Bartra Joan, Bobolea Irina, Campo Paloma, Cardona Vicky, Carriazo Ana Maria, Castillo Vizuete José Antonio, Chivato Tomas, Davila Gonzalez Ignacio Jesus, Mullol Joaquim, Nieto Garcia Antonio, Picado César, Quirce Santiago, Sastre Joaquin, de las Vecillas Leticia from Spain, Benson Mikael, Kull Inger, van Hage Marianne, Wickman Magnus from Sweden, Khaltaev Nikolai from Switzerland, Mohammad Yousser from Syria, Tantilipikorn Pongsakorn, Vichyanond Pakit from Thailand, Cingi Cemal, Gemicioglu Bilun, Goksel Ozlem, Kalayci Omer, Kalyoncu Ali Fuat, Meço Cem, Vardaloglu Koyuncu Ilgim, Yorgancioglu Arzu, Karabacak Deniz Eyice from Turkey, Kirenga Bruce from Uganda, Adcock Ian Michael, Arshad Hasan S, Bewick Mike, Bond Christine, Brightling Christopher, Bush Andrew, Calderon Moïses, Chung Kian Fan, Custovic Adnan, Djukanovic Ratko, Durham Stephen, Farrell John, Halpin David, Hyland Michael, Johnston Sebastian, Lipworth Brian, Nakonechna Alla, Price David, Roberts Graham, Ryan Dermot, Schwarze Jürgen, Sheikh Aziz, Shields Mike, Walker Samantha, Williams Sian from United Kingdom, Kaidashev Igor, Kurchenko Andrii Igorevich, Tsaryk Vladyslav from Ukraine, Bernstein David, Bernstein Jonathan A, Casale Thomas B, Chinthrajah Sharon, Dykewicz Mark, Marshall Gailen D, Meltzer Eli O, Naclerio Robert M, Togias Alkis, Toskala Elina, Wallace Dana, Williams Dennis M, Yawn Barbara from United States, Caballero-Fonseca Fernan from Venezuela, Le Thi Tuyet Lan, Vu Tran Thien Quan from Vietnam.
