# Supplementary material for: Perceptions of the impact of individual allergic rhinitis symptoms: A survey of ARIA clinical experts
Source: World Allergy Organ J. 2024 Dec 3;17(12):100999. doi: 10.1016/j.waojou.2024.100999 (PMC11665388; doi:10.1016/j.waojou.2024.100999)
Supplement: Multimedia component 1 [file mmc1.docx]

**SUPPLEMENTAL TABLE 1:** Symptoms most frequently reported by patients and perceived as the most bothersome, as stated by ARIA physicians who see patients with AR, according to respondents’ status in relation to AR.

|  |  | 1. **Patients’ most frequent symptoms – N (%)** | | | |  |
| --- | --- | --- | --- | --- | --- | --- |
|  |  | **Nasal** | **Ocular** | **Asthma** | **Other** | **p-value** |
| **Suffers from AR** | **Yes** | 128 (94.8) | 3 (2.2) | 4 (3.0) | 0 (0.0) | 0.821 |
|  | **No** | 188 (96.0) | 4 (2.0) | 3 (1.5) | 1 (0.5) |  |
|  | **Prefers not to say** | 9 (100.0) | 0 (0.0) | 0 (0.0) | 0 (0.0) |  |
|  |  | 1. **Patients’ most bothersome symptoms – N (%)** | | | |  |
|  |  | **Nasal** | **Ocular** | **Asthma** | **Other** | **p-value** |
| **Suffers from AR** | **Yes** | 77 (57.0) | 22 (16.3) | 34 (25.2) | 2 (1.5) | 0.162 |
|  | **No** | 133 (67.9) | 19 (9.7) | 41 (21.0) | 3 (1.5) |  |
|  | **Prefers not to say** | 5 (55.6) | 0 (0.0) | 4 (44.4) | 0 (0.0) |  |
|  |  | **C. Patients’ most bothersome nasal symptoms – N (%)** | | | |  |
|  |  | **Congestion** | **Rhinorrhea** | **Itching** | **Sneezing** | **p-value** |
| **Suffers from AR** | **Yes** | 113 (83.7) | 19 (14.1) | 2 (1.5) | 1 (0.7) | 0.534 |
|  | **No** | 161 (82.1) | 24 (12.2) | 6 (3.1) | 5 (2.6) |  |
|  | **Prefers not to say** | 4 (44.4) | 4 (44.4) | 0 (0.0) | 1 (11.1) |  |

**SUPPLEMENTAL TABLE 2:** Most bothersome ARIA members’ symptoms according to age group and sex, and their perception of patients’ most bothersome symptoms

|  |  | **ARIA member’s most bothersome symptoms – N (%)** | | | |  |
| --- | --- | --- | --- | --- | --- | --- |
|  |  | | **Nasal** | **Ocular** | **Asthma** | **p-value** |
| **Age group** | **18-35 years** | | 4 (2.6) | 1 (0.6) | 0 (0.0) | 0.941 |
|  | **36-50 years** | | 28 (18.1) | 7 (4.5) | 0 (0.0) |  |
|  | **51-65 years** | | 62 (40.0) | 13 (8.4) | 4 (2.6) |  |
|  | **66+ years** | | 26 (16.8) | 5 (3.2) | 1 (0.6) |  |
|  | **Not answered** | | 4 (2.6) | 0 (0.0) | 0 (0.0) |  |
| **Sex** | **Female** | | 39 (25.2) | 10 (6.5) | 2 (1.3) | 0.618 |
|  | **Male** | | 85 (54.8) | 16 (10.3) | 3 (1.9) |  |
| **Patients’ most bothersome symptoms** | **Nasal** | | 69 (51.1) | 8 (5.9) | 0 (0.0) | 0.001 |
|  | **Ocular** | | 13 (9.6) | 8 (5.9) | 1 (0.7) |  |
|  | **Asthma** | | 27 (20.0) | 5 (3.7) | 2 (1.5) |  |
|  | **Other symptoms** | | 1 (0.7) | 0 (0.0) | 1 (0.7) |  |

**SUPPLEMENTAL TABLE 3:** Most frequent ARIA members’ symptoms according to age group, sex and their perception of patients’ most frequent symptoms

|  |  |  |  |  |  |  |
| --- | --- | --- | --- | --- | --- | --- |
|  |  | **ARIA member’s most frequent symptoms – N (%)** | | | |  |
|  |  | **Nasal** | **Ocular** | **Asthma** | **Other** | **p-value** |
| **Age group** | **18-35 years** | 4 (2.6) | 0 (0.0) | 0 (0.0) | 0 (0.0) | 0.416 |
|  | **36-50 years** | 34 (21.9) | 1 (0.6) | 0 (0.0) | 0 (0.0) |  |
|  | **51-65 years** | 71 (45.8) | 7 (4.5) | 1 (0.6) | 0 (0.0) |  |
|  | **66+ years** | 26 (16.8) | 4 (2.6) | 1 (0.6) | 1 (0.6) |  |
|  | **Not answered** | 4 (2.6) | 0 (0.0) | 0 (0.0) | 0 (0.0) |  |
| **Sex** | **Female** | 43 (27.7) | 6 (3.9) | 2 (1.3) | - | 0.123 |
|  | **Male** | 96 (61.9) | 7 (4.5) | 0 (0.0) | - |  |
| **Patients’ most frequent symptoms** | **Nasal** | 118 (87.4) | 8 (5.9) | 1 (0.7) | 1 (0.7) | 0.290 |
|  | **Ocular** | 2 (1.5) | 1 (0.7) | 0 (0.0) | 0 (0.0) |  |
|  | **Asthma** | 4 (3.0) | 0 (0.0) | 0 (0.0) | 0 (0.0) |  |
|  | **Other symptoms** | - | - | - | - |  |

**SUPPLEMENTAL BOX 1:** Survey sent by email to ARIA members; * represents mandatory questions to its completion.

| Survey: Impairment in Allergic Rhinitis  1. About you  The following questions gather some information about you. By providing your e-mail address, you may participate as a group co-author in future publications.  1.1. Please select your country. *  1.2. What is your sex? *  1.3. What is your age?  1.4. Which of the following describe your medical specialty? *   - Allergy/Allergology - General Practice/Primary Care Internal Medicine Otorhinolaryngology (ENT Physician) Pediatrics - Pulmonology - I am not a physician - Other   1.5. For how many years have you been in clinical practice? *  2. Are you a physician who sees patients with allergic rhinitis in your clinical practice? *   - Yes. I see only adult patients with allergic rhinitis - Yes. I see only pediatric patients with allergic rhinitis - Yes. I see both adult and pediatric patients with allergic rhinitis - No, I don't see patients with allergic rhinitis   3. Assessment of allergic rhinitis symptoms among your patients  The following questions now aim to assess the perception you have on the most bothersome allergic rhinitis symptoms among your patients with allergic rhinitis  3.1. Overall, what are the symptoms most frequently reported by your patients with allergic rhinitis? *   - Ocular symptoms - Nasal symptoms - Asthma symptoms - Other symptoms (non-respiratory and non-ocular symptoms)   3.2. In your perception, what are the symptoms that, when present, are the most bothersome to your patients with allergic rhinitis? *   - Ocular symptoms - Nasal symptoms - Asthma symptoms - Other symptoms (non-respiratory and non-ocular symptoms)   3.3. In your perception, what are the nasal symptoms that, when present, are the most bothersome to your patients with allergic rhinitis? *   - Nasal congestion - Nasal itching - Rhinorrhea - Sneezing   4. Assessment of your experience as an allergic rhinitis patient  The following questions now aim to assess what are the most bothersome allergic rhinitis symptoms for you as a patient.  4.1. Do you have allergic rhinitis? *   - Yes - No - Prefer not to say   4.2. Considering the ARIA classification, which of the following options best describes your allergic rhinitis? *   - Mild intermittent - Mild persistent - Moderate-severe intermittent - Moderate-severe persistent   4.3. What are the most frequent allergic rhinitis symptoms you have? *   - Nasal symptoms (nasal congestion, itching, rhinorrhea and/or sneezing) - Ocular symptoms (ocular redness, itching and/or tearing/watering) - Asthma symptoms - Other   4.4. What are the allergic rhinitis symptoms that, when present, bother you the most? *   - Nasal symptoms (nasal congestion, itching, rhinorrhea and/or sneezing) - Ocular symptoms (ocular redness, itching and/or tearing/watering) - Asthma symptoms - Other   4.5. Which of the following nasal symptoms bother you the most, when present? *   - Nasal congestion - Nasal itching - Rhinorrhea - Sneezing   5. If you have any comments or observations before submitting the survey, please leave them here. |
| --- |
